# Measurement Uncertainty, Purity, and Entanglement Dynamics of Maximally Entangled Two Qubits Interacting Spatially with Isolated Cavities: Intrinsic Decoherence Effect

**DOI:** 10.3390/e24040545

**Published:** 2022-04-13

**Authors:** Abdel-Baset A. Mohamed, Atta Ur Rahman, Hichem Eleuch

**Affiliations:** 1 Department of Mathematics, College of Science and Humanities in Al-Aflaj, Prince Sattam bin Abdulaziz University, Al-Kharj 11942, Saudi Arabia; 2 Department of Mathematics, Faculty of Science, Assiut University, Assiut 71515, Egypt; 3 Key Laboratory of Aerospace Information Security and Trusted Computing, Wuhan University, Wuhan 430072, China; attapk@outlook.com; 4 Department of Applied Physics and Astronomy, University of Sharjah, Sharjah P.O. Box 26666, United Arab Emirates; hichemeleuch@yahoo.fr; 5 College of Arts and Sciences, Abu Dhabi University, Abu Dhabi P.O. Box 59911, United Arab Emirates; 6 Institute for Quantum Science and Engineering, Texas A&M University, College Station, TX 77843, USA

**Keywords:** quantum memory-assisted entropic uncertainty, purity, entanglement

## Abstract

In a system of two charge-qubits that are initially prepared in a maximally entangled Bell’s state, the dynamics of quantum memory-assisted entropic uncertainty, purity, and negative entanglement are investigated. Isolated external cavity fields are considered in two different configurations: coherent-even coherent and even coherent cavity fields. For different initial cavity configurations, the temporal evolution of the final state of qubits and cavities is solved analytically. The effects of intrinsic decoherence and detuning strength on the dynamics of bipartite entropic uncertainty, purity and entanglement are explored. Depending on the field parameters, nonclassical correlations can be preserved. Nonclassical correlations and revival aspects appear to be significantly inhibited when intrinsic decoherence increases. Nonclassical correlations stay longer and have greater revivals due to the high detuning of the two qubits and the coherence strength of the initial cavity fields. Quantum memory-assisted entropic uncertainty and entropy have similar dynamics while the negativity presents fewer revivals in contrast.

## 1. Introduction

Recently, several quantum computing studies have focused on superconducting (SC) circuits based on Josephson junctions because they can be relatively easily implemented as qubits [1,2]. Despite the fact that the normal decoherence durations of these circuits are significantly less than what is necessary for quantum computation, their macroscopic quantum coherence is sufficient for them to demonstrate spectacular quantum behaviors [3,4,5,6]. Many SC eigenstates with quantized eigenvalues may exist in such circuits [7]. Because of this characteristic, these circuits can function as artificial SC atoms. Artificial atoms are generated by using SC circuits which resemble natural atoms substantially [8]. Unlike natural atoms, artificial atoms may be produced with defined properties and features on-chip using normal lithographic technologies [9]. This level of adaptability is considered beneficial in terms of applicability. SC circuits may therefore illustrate quantum optics, information processing, and associated protocols on a chip in a controlled way, as well as exhibit key quantum mechanical concepts on a macro-scale [10,11,12,13].

It is also possible to create artificial atoms having features that do not exist in natural atoms. Condensed electrons are used in SC circuits to take advantage of the inherent coherence of the SC state. SC electrons can be employed to encode non-local information as charge-qubits, flux-qubits, or phase-qubits [14,15]. The systems, which work at temperatures below 100 mK, are usually built using thin-film technology. On-chip types of equipment are employed to perform the measurements. These types of chips are based on Josephson junctions [14], which are currently the most experimentally advanced chip devices comprising solid-state qubits. The charge qubit can be controlled flexibly through external tunable parameters, so it serves as an operational qubit [15,16].

The uncertainty principle is considered one of the most fundamental notions in quantum physics. Heisenberg [17] was the first to propose the well-known uncertainty principle [18]. Later, Kennard proved this for a particle [19]. Robertson developed a generalized formula based on a modification of the Heisenberg uncertainty relation when working with any two non-commuting observables [20]. Robertson’s lower bound inequality is independent of the system state when the system is prepared in the eigenstates of either of the two observables [20]. The concept of entropy was expected to be useful in describing the uncertainty in quantum information theory. Everett and Hirschman were the first to suggest an entropy-based uncertainty relation for position and momentum observables [21]. In recent years, cryptography [22], witness of entanglement [23], quantum-speed limit [24], quantum-key distributions [25], and quantum metrology [26] have all been recognized as major applications of quantum memory entropic uncertainty relations in quantum information processing.

Quantum physics and quantum technologies are built on the concept of entanglement [27,28,29]. Like other aspects of quantum physics, this nonlocal correlation is realized at extremely small scales. When two particles, such as photons or electrons, become entangled, they stay linked even though they are separated by huge distances. This unique physical feature presents a notable variety of applications in quantum information and quantum technology. Super-dense coding [30], quantum communication [31], teleportation [32,33], and computation [34], quantum private comparison [35] and other processes of quantum processing rely on entanglement for their effective practical deployment. Entanglement is a phenomenon that could reduce the time and processing power required to manage data flow between qubits. The capacity of a quantum state to preserve entanglement and superposition is referred to as quantum coherence [36]. Quantum coherence is essential in quantum information sciences, quantum biology, and quantum thermodynamics, as well as physics. Coherence can be considered as a resource similar to entanglement but significantly more fundamental [37]. Here, we take two qubits and expose them to coherent cavity fields and analyze the quantum correlations’ dynamics. The activity of coherent fields can significantly improve the efficiency of quantum information processing tasks. The imposed fields and their effects on the dynamics of quantum systems may reveal a variety of effects. We find that different coupled field properties can significantly alter the delayed preservation of nonclassical correlations in quantum systems. When the transmitting media are classically correlated, the resulting quantum correlations become less susceptible to the external noise [38]. In Ref. [39], the authors demonstrated that imposing a nuclear bath on the dynamics of electrons in gate-defined GaAs quantum dots leads to a shorter dephasing time. Many additional research works have reached the same result, namely that different types of coupled fields have different properties for sustaining quantum correlations [40,41,42]. This inspires us to prepare the coupled coherent fields in two variants: coherent-even coherent and coherent-even coherent cavity fields. In the first case, we combine a coherent and even coherent field and apply it to the two-qubit dynamics. In the latter case, the system is prepared with two even coherent state fields. This allow us to identify the characteristics of various coherent fields and their effects on the preservation of nonclassical correlations.

Quantum correlations are vulnerable to decoherence effects, and, as a result, quantum systems cannot be protected from decoherence. Even in closed quantum systems, decoherence occurs, such as the intrinsic decoherence [43], which has been studied in various closed qubit systems [44,45]. Therefore, in this work, we are motivated to investigate the dynamics of entangled two charge-qubits, where each qubit interacts with an isolated external cavity field under intrinsic decoherence. As previously mentioned, charge qubits are among the most reliable resources for quantum information and other related applications. Here, we focus on the coupled cavities’ ability to alter nonclassical correlations, entropic uncertainty and entropy purity. The generated quantum information resources are quantified using two-charge-qubit quantum memory-assisted entropic uncertainty, entropy, and negativity. The effects of the system parameters and initial states on the quantum information resources are discussed. Furthermore, we will relate the rate of quantum correlations and purity degradation to the memory effects in the coherent fields. Karpat et al. demonstrated that there is a relationship between the rate of entropic uncertainty and varied values of memory effects [46]. Using the interpretation in Ref. [46], we will associate various elements of the coherent fields and system to memory properties.

The following is the breakdown of the paper’s content. The physical model and its dynamics are introduced in Section 2. In Section 3, we focus on quantum information resources. Section 4 discusses the results for various initial charge-qubit states. Finally, in Section 5, we provide our conclusions.

## 2. The Physical Model and Its Dynamics

### 2.1. Physical Description

In this section, we provide the details of the dynamics of quantum memory-assisted entropic uncertainty, entropy, and the negativity of maximally entangled two charge-qubits (*A* and *B*). Each one of them interacts spatially with an isolated coherent *i*-cavity (i=A,B) field with frequency ω. The *i*—charge-qubit Cooper-pair-box is engineered by: (1) a tiny superconducting island linked to two identical Josephson junctions that are controlled by the same capacitance $C_{J}^{i}$ and energy $E_{J}^{i}$; (2) a gate voltage $V_{g}^{i}$ coupled to a gate capacitor $Ci_{g}^{i}$ with the dimensionless gate charge $n_{g}^{i}=C_{g}^{i}V_{g}^{i}/2e$. This Cooper-pair-box works experimentally [47] as a charge qubit when $k_{B}^{i}T^{i}\ll E_{J}^{i}\ll E_{C}^{i}\ll ▵$ (Boltzmann constant $k_{B}^{i}$, temperature $T^{i}$, charge $E_{C}^{i}$, and gap energies *▵*). When the gate voltage range is near a degeneracy point, $n_{g}^{i}=\frac{1}{2}$, and the charge-qubit can be considered as only having upper $|1_{i}\rangle$ and lower $|0_{i}\rangle$ states, and the other charge states can be neglected. The qubit–photon interaction is controlled by the classical flux $\Phi_{c}^{i}$, $n_{g}^{i}$, and the magnetic flux $\Phi_{0}^{i}$, where $\Phi_{c}^{i}=\Phi_{0}^{i}/2$. The charge-qubit–cavity detuning frequencies are: $\delta_{i}=\frac{e}{C_{s}^{i}}\left(C_{g}^{i}V_{g}^{i}-e\right)-\omega_{i}$ with $C_{s}=C_{g}^{i}+2C_{J}^{i}$. Therefore, in the rotating wave approximation, the charge-qubit–cavity Hamiltonian is given by [48],

(1) $$\begin{array}{ccc} \hat{H} & = & \sum_{i=A,B}\omega_{i}{\hat{\psi }}_{i}^{†}{\hat{\psi }}_{i}+\left(\delta_{i}+\omega_{i}\right){\hat{\sigma }}_{z}^{i}+\frac{\pi \eta^{i}E_{J}^{i}}{\Phi_{0}^{i}}\left({\hat{\psi }}_{i}{\hat{\sigma }}_{+}^{i}+{\hat{\psi }}_{i}^{†}{\hat{\sigma }}_{-}^{i}\right), \end{array}$$

where ${\hat{\psi }}_{i}^{†}\left({\hat{\psi }}_{i}\right)$ are the creation and annihilation operators of the *i*-cavity. ${\hat{\sigma }}_{z}$ and ${\hat{\sigma }}_{x}$ are the *i*—charge-qubit operators. The parameter $\eta^{i}$ is in the unit of magnetic flux and depends on the engineering properties of the cavity and the *i*—charge-qubit Cooper-pair-box.

We focus here on the effect of intrinsic decoherence. In the presence of the intrinsic decoherence, the system evolution is governed by a stochastic sequence of identical unitary transformations rather than a continuous unitary evolution [43]. Therefore, the dynamics of the qubit–cavity system is governed by the Milburn equation [43]

(2) $$\frac{d}{dt}\hat{M}\left(t\right)=-i\left[H,\hat{M}\right]-\frac{\gamma }{2}\left[H,\left[H,\hat{M}\right]\right],$$

where γ is the decoherence parameter, and $\hat{M}\left(t\right)$ represents the temporal qubit–cavity density matrix.

### 2.2. The Solution of the Milburn Equation

To investigate the dynamics of the two-charge-qubit quantum memory-assisted entropic uncertainty, entropy as well as the negativity, we assume that the initial two-charge-qubit state is asymmetric Bell states $|\psi_{AB}\left(0\right)\rangle$,

(3) $$\begin{array}{ccc} |\Psi_{AB}\left(0\right)\rangle & = & \frac{1}{\sqrt{2}}(|1_{A}0_{B}\rangle -|0_{A}1_{B}\rangle ). \end{array}$$

The two cavities are prepared initially in two different cases. In the first case, we present an initial, coherent-even coherent (CEC) cavity state. In CEC configuration, one of the cavities is constructed as a coherent state, $|\alpha_{i}^{C}\rangle$, i=A,B, and another is an even coherent state $|\alpha_{i}^{EC}\rangle =(|\alpha_{i}\rangle +|-\alpha_{i}\rangle )/A$, where $|\alpha_{i}\rangle =\sum_{n}\eta_{n}{|n\rangle }_{i},\eta_{n}=e^{-\frac{1}{2}N_{i}^{2}}\frac{\alpha_{i}^{n}}{\sqrt{n!}}$, $N_{i}={\left|\alpha_{i}\right|}^{2}$ is the amplitude of the coherent states (mean photon numbers), and *n* is the number of photons inside the cavity. In the latter case, the two cavities are in even coherent (EC) states $|\alpha_{i}^{EC}\rangle$, i=A,B.

By using the asymmetric Bell state and the eigenvectors $|\varphi_{n}^{\pm }\rangle_{i}=\frac{1}{\sqrt{2}}{(|1_{i},n\rangle }_{i}\pm |0_{i},n+1\rangle_{i})$ of the *i*-cavity–qubit Hamiltonian (i=A,B), we intend to find a particular solution of the two-charge-qubit system.

When the two charge-qubits have an initial asymmetric Bell state, while the two cavities have one of the considered initial coherent states, the total initial cavity–qubit state can be written as:

(4) $$\begin{array}{ccc} \hat{M}\left(0\right) & = & \frac{1}{2}\left[\Lambda_{1}\left(0\right)-\Lambda_{2}\left(0\right)-\Lambda_{3}\left(0\right)+\Lambda_{4}\left(0\right)\right]. \end{array}$$

where

(5) $$\begin{array}{ccc} \;\;\;\;\;\;\;\;\;\;\;\;\;\;\;\Lambda_{1}\left(0\right) & = & |1_{A},\alpha_{A}^{i}\rangle \langle 1_{A},\alpha_{A}^{i}\left|⊗\right|0_{B},\alpha_{B}^{i}\rangle \langle 0_{B},\alpha_{B}^{i}|,\;\\ \;\;\;\;\;\;\;\;\;\;\;\;\;\;\;\Lambda_{2}\left(0\right) & = & |0_{A},\alpha_{A}^{i}\rangle \langle 1_{A},\alpha_{A}^{i}\left|⊗\right|1_{B},\alpha_{B}^{i}\rangle \langle 0_{B},\alpha_{B}^{i}|,\;\\ \;\;\;\;\;\;\;\;\;\;\;\;\;\;\;\Lambda_{3}\left(0\right) & = & |1_{A},\alpha_{A}^{i}\rangle \langle 0_{A},\alpha_{A}^{i}\left|⊗\right|0_{B},\alpha_{B}^{i}\rangle \langle 1_{B},\alpha_{B}^{i}|,\;\\ \;\;\;\;\;\;\;\;\;\;\;\;\;\;\;\Lambda_{4}\left(0\right) & = & |0_{A},\alpha_{A}^{i}\rangle \langle 0_{A},\alpha_{A}^{i}\left|⊗\right|1_{B},\alpha_{B}^{i}\rangle \langle 1_{B},\alpha_{B}^{i}|. \end{array}$$

After expressing the initial state (4) in terms of the dressed cavity–qubit state, $|\varphi_{n}^{\pm }\rangle_{i}$ of the Hamiltonian (1). We can derive the time evolution of the cavity–qubit system. Explicitly,

(6) $$\begin{array}{ccc} \hat{M}\left(t\right) & = & \frac{1}{2}[\Lambda_{A}^{11}\left(t\right)⊗\Lambda_{B}^{00}\left(t\right)-\Lambda_{A}^{10}\left(t\right)⊗\Lambda_{B}^{01}\left(t\right)\;\\ \; & \; & -\Lambda_{A}^{01}\left(t\right)⊗\Lambda_{B}^{10}\left(t\right)+\Lambda_{A}^{00}\left(t\right)⊗\Lambda_{B}^{11}\left(t\right)]. \end{array}$$

where $\Lambda_{i}^{kl}\left(t\right)$ are given by

$$\begin{array}{ccc} \Lambda_{i}^{11}\left(t\right) & = & \frac{1}{4}\sum_{m,n=0}\eta_{m,n}(\lambda_{1}|1_{i},m\rangle \langle 1_{i},n|+\lambda_{2}|1_{i},m\rangle \langle 0_{i},\bar{n}|\\ & & \;+\lambda_{3}|0_{i},\bar{m}\rangle \langle 1_{i},n|+\lambda_{4}|0_{i},\bar{m}\rangle \langle 0_{i},\bar{n}|),\\ \Lambda_{i}^{00}\left(t\right) & = & e^{-N_{i}^{2}}|0_{i},0\rangle \langle 0_{i},0|+[\sum_{m=0}\eta_{\bar{m},0}e^{-\frac{1}{2\gamma }\nu_{m}^{2}t}[-i\sin\nu_{m}t\\ & & \times |1_{i},m\rangle \langle 0_{i},0|+\cos\nu_{m}t|0_{i},\bar{m}\rangle \langle 0_{i},0|]+h.c.]\\ & + & \frac{1}{4}\sum_{m,n=0}\eta_{\bar{m},\bar{n}}(\lambda_{4}|1_{i},m\rangle \langle 1_{i},n|+\lambda_{3}|1_{i},m\rangle \langle 0_{i},\bar{n}|\\ & + & \lambda_{2}|0_{i},\bar{m}\rangle \langle 1_{i},n|+\lambda_{1}|0_{i},\bar{m}\rangle \langle 0_{i},\bar{n}|),\\ \Lambda_{i}^{10}\left(t\right) & = & \sum_{m=0}\eta_{m,0}e^{-\frac{1}{2\gamma }\nu_{m}^{2}t}(\cos\nu_{m}t|1_{i},m\rangle \langle 0_{i},0|-i\sin\nu_{m}t\\ & \times & |0_{i},\bar{m}\rangle \langle 0_{i},0|)+\frac{1}{4}\sum_{m,n=0}\eta_{m,\bar{n}}(\lambda_{2}|1_{i},m\rangle \langle 1_{i},n|+\\ & \lambda_{1} & \;|1_{i},m\rangle \langle 0_{i},\bar{n}|\;+\;\lambda_{4}|0_{i},\bar{m}\rangle \langle 1_{i},n|\;+\;\lambda_{3}|0_{i},\bar{m}\rangle \langle 0_{i},\bar{n}|), \end{array}$$

with:

$$\begin{array}{ccc} \lambda_{1} & = & R_{1}+R_{2}+R_{3}+R_{4},\lambda_{2}=R_{1}-R_{2}+R_{3}-R_{4},\\ \lambda_{3} & = & R_{1}+R_{2}-R_{3}-R_{4},\lambda_{4}=R_{1}-R_{2}-R_{3}+R_{4},\\ R_{i} & = & e^{-i\beta_{i}t-\frac{1}{2\gamma }\beta_{i}^{2}t},\eta_{mn}=\eta_{m}\eta_{n}^{*},\bar{m}=m+1,\\ \nu_{m}^{i} & = & \lambda_{i}\sqrt{m+1},\;\;\beta_{1}=\varepsilon_{m}^{+}-\varepsilon_{n}^{+},\;\;\beta_{2}=\varepsilon_{m}^{+}-\varepsilon_{n}^{-},\\ \beta_{3} & = & \varepsilon_{m}^{-}-\varepsilon_{n}^{+},\;\;\beta_{4}=\varepsilon_{m}^{-}-\varepsilon_{n}^{-}, \end{array}$$

where $\varepsilon_{i}^{\pm }$ are the eigenvalues of the *i*-cavity–qubit Hamiltonian (i=A,B). If we replace $1_{A}$ by $1_{B}$ and $0_{A}$ by $0_{B}$, we obtain the elements $\Lambda_{B}^{kl}\left(t\right)$. To investigate the dynamics of the two-charge-qubit quantum memory and coherence, we find the two-charge-qubit density matrix by tracing out the coherent cavity state $|m_{A}\rangle ⊗|n_{B}\rangle$ from the final cavity–qubit state of Equation (6). Then, the determined two-charge-qubit state is given by:

(7) $$\begin{array}{c} {\hat{M}}_{AB}\left(t\right)=\;\;\;\;\;\;\sum_{m_{A},n_{B}=0}^{\infty }\sum_{k,l=1,0}\;\;\;\frac{{\left(-1\right)}^{k+l}}{2}T_{A}^{kl}\left(m\right)⊗T_{B}^{lk}\left(n\right), \end{array}$$

$T_{A}^{kl}\left(m\right)=\langle m_{A}|\Lambda_{A}^{kl}\left(t\right)|m_{A}\rangle$. The above two-charge-qubit reduced density matrices are used to quantify the dynamics of the two-charge-qubit quantum memory and coherence using the entropic uncertainty, entropy and negativity entanglement as measures.

## 3. Quantum Information Resources Measures

The two-charge-qubit system is initially prepared in maximally correlated states. The dynamics of the maximal initial two-qubit quantum memory-assisted entropic uncertainty, entropy purity and negativity are used as measures to study the quantum correlations of the system. They are defined as follows:

- **Entropic uncertainty**
  For incompatible observables *P* and *Q*, Bob’s uncertainty regarding the two qubits (*A* and *B*) measurement outcome is given by [49,50]:
  (8) $$S\left(P|B\right)+S\left(Q|B\right)\geq S\left(A|B\right)+\log_{2}\frac{1}{c},$$
  where $S\left(A|B\right)=S\left({\hat{M}}_{AB}\right)-S\left({\hat{M}}_{B}\right)$ represents the ${\hat{M}}_{AB}$ operator’s conditional von Neumann entropy with $S\left(\hat{M}\right)=-tr\left(\hat{M}\log_{2}\hat{M}\right)$ (for a density matrix $\hat{M}$). $S\left(X|B\right)=S\left({\hat{M}}^{XB}\right)-S\left({\hat{M}}_{B}\right)$, X∈{P,Q} is the post measurement state ${\hat{R}}^{XB}=\sum_{x}(|\psi_{x}\rangle \langle \psi_{x}|⊗\hat{I}){\hat{M}}_{AB}\left(|\psi_{x}\rangle \langle \psi_{x}|⊗I\right)$. Here, ${\hat{M}}_{B}=tr_{A}\left({\hat{M}}^{AB}\right)$ and $|\psi_{x}\rangle$ designs the eigenvectors of *X*. $\hat{I}$ is the identical operator. The left UL and right UR entropic uncertainty sides of Equation (8) can be represented as follows:
  (9) $$\begin{array}{cc} UL(t) & =S\left({\hat{M}}_{\sigma_{x}B}\right)+S\left({\hat{M}}_{\sigma_{z}B}\right)-2S\left({\hat{M}}_{B}\right), \end{array}$$
  (10) $$\begin{array}{cc} UR(t) & =S\left({\hat{M}}_{AB}\right)-S\left({\hat{M}}_{B}\right)+1, \end{array}$$
  where L(t) and R(t) are, respectively, the entropic uncertainty and its lower bound.
- **Two-charge-qubit entropy purity (EP)**
  Here, entropy is used to quantify the amount of two-charge-qubit purity/mixedness [51].
  The qubit–qubit entropy is defined by:
  (11) $$\begin{array}{c} EP\left(t\right)=-\sum_{i=1}^{\infty }\lambda^{i}\;\;\ln\left(\lambda^{i}\right), \end{array}$$
  which depends on the eigenvalues $\lambda^{i}$ of the two-charge-qubit state ${\hat{M}}_{AB}\left(t\right)$.
- **Two-qubit negativity entanglement (NE)**:
  The negativity is a good entanglement monotonic measure. In the current case, NE(t) is used to investigate the two-charge-qubit entanglement [52]. It is equal to the absolute sum of the negative eigenvalues of the density matrix ${\left({\hat{M}}_{AB}\left(t\right)\right)}^{T_{A}}$ that is the partial transpose of the two-charge-qubit density matrix ${\hat{M}}_{AB}$ with respect to subsystem *A*. The elements of ${\left({\hat{R}}^{AB}\right)}^{T_{A}}$ are given by:
  (12) $$\begin{array}{c} \langle i,j|{\left({\hat{M}}_{AB}\left(t\right)\right)}^{T_{A}}|m,n\rangle =\langle m,j|{\hat{M}}_{AB}\left(t\right)|i,n\rangle . \end{array}$$
  When NE(t)=0, the state is separable. The function NE(t) is used to estimate the entanglement amount of the quantum state.

## 4. Discussion

The impact of intrinsic decoherence and qubit–cavity interaction parameters on the dynamics of quantum memory-assisted entropic uncertainty (UR(t) and UL(t)) given in Equations (9) and (), entropy purity EP(t) in Equation (11), and negativity entanglement NE(t) in Equation (12) are investigated in this section. Here, we assume that the two-charge-qubit system is initially in the maximally correlated asymmetric Bell state, $|\Psi_{AB}^{S}\left(0\right)\rangle =\frac{1}{\sqrt{2}}(|1_{A}0_{B}\rangle -|0_{A}1_{B}\rangle )$. For this initial maximally correlated state, the initial entropic uncertainty’ values are UR(0)=UL(0)=0 and the initial entropy EP(0)=0, while the negativity is NE(0)=1.

**Figure 1:**
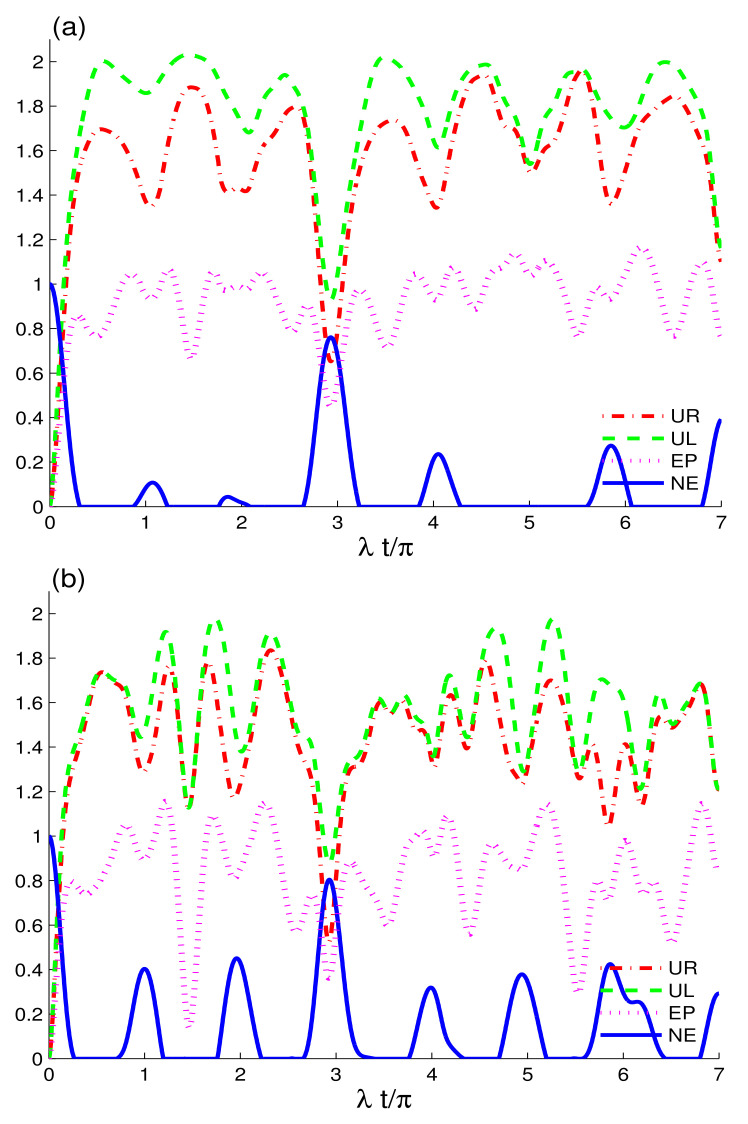
Dynamics of the quantum memory-assisted entropic uncertainty (UR(t) and UL(t)), entropy purity EP(t), and negativity entanglement NE(t) are shown for the initial maximally correlated state $\frac{1}{\sqrt{2}}(|1_{A}0_{B}\rangle -|0_{A}1_{B}\rangle )$ in the absence of the decoherence γ=0 and detunings $\delta_{i}=0$. When the cavities are initially in CEC cavity state in (**a**) and in EC cavity state in (**b**) for small coherent strengths, $|\alpha_{A}{|}^{2}=0.9$ and $|\alpha_{B}{|}^{2}=0.5$.

Figure 1 analyzes the time evolution of entropic uncertainty, purity and entanglement in two non-interacting qubits initially prepared in the maximally entangled state coupled to CEC (a) and EC configuration (b), while the amplitude of the coherent state is: $|\alpha_{A}{|}^{2}=0.9$ and $|\alpha_{B}{|}^{2}=0.5$. We investigate the effects of the electromagnetic fields in the absence of intrinsic decoherence and two-charge-qubit detuning. UL(t) and UR(t) have different dynamical behaviours, and UL(t)>UR(t). Entropic uncertainty functions, UL(t), UR(t), and EP(t) grow with time, whereas NE(t) shows a decline. The increase in entropic uncertainty indicates the formation of temporal quantum memory, which is in concordance with the results in [46]. This demonstrates that the loss of purity and entanglement is caused by the rise in entropic uncertainty between the charge-qubits and fields. As can be observed in Figure 1a, the two-charge qubits are initially maximally correlated. However, the correlations are reduced when the interaction between the qubits and fields, both in CEC and EC cavities, is switched on. The decrease in correlations between the two qubits is caused by the interaction of the qubits with the coupled fields and not by the intrinsic decoherence, as γ=0. In comparison to the initial correlations, we deduce that the correlations and purity of the two qubits face sudden death, as shown by NE(t) and EP(t). The initial correlations, despite being permanently lost, decrease temporarily and, hereafter, the nonlocal correlations and purity are constantly reappearing. This indicates that, due to the formation of entropic uncertainty, mixedness in the system grows. When it reduces, the state regains the order, enhancing the purity of the state. We observe that, in CEC and EC configurations, the preservation of nonlocal correlations and information exchange between charge-qubits and coupled fields is not the same. When compared to the CEC configuration, the EC configuration preserves more nonlocal correlations as well as exhibits a better revival function. As time evolves, the EC configuration increases the width of nonlocal correlations revivals. The robustness of the quantum correlations in the current case depends on the CEC and EC configurations.

**Figure 2:**
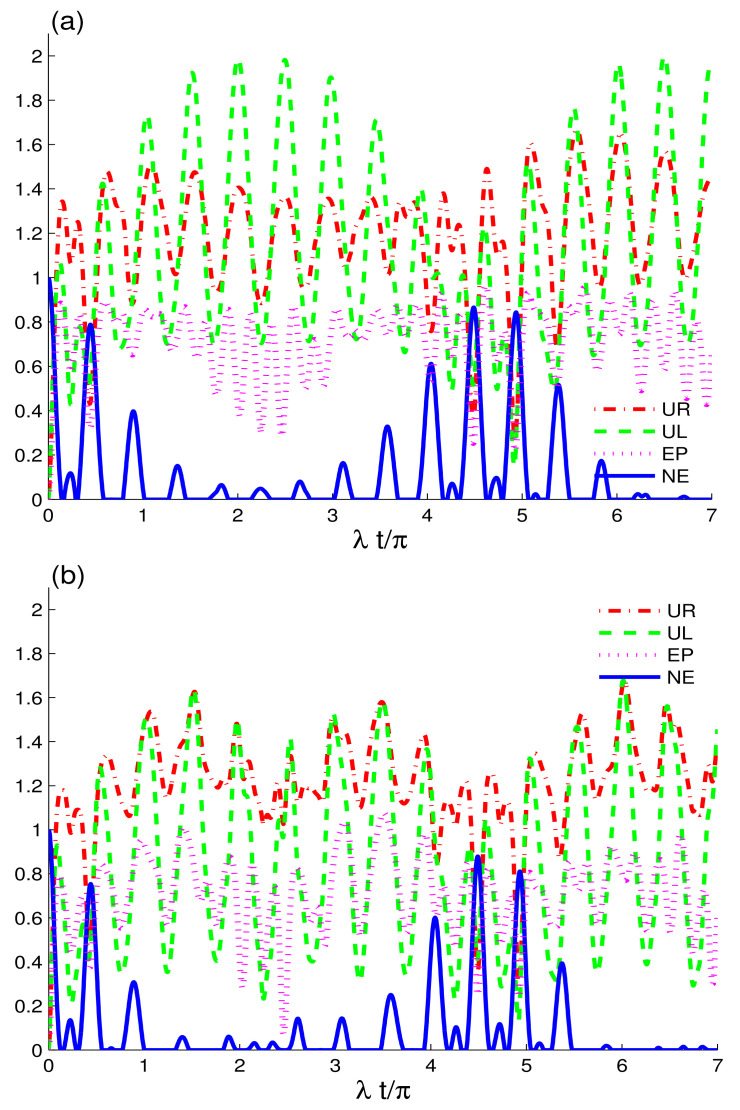
Dynamics of the quantum memory-assisted entropic uncertainty (UR(t) and UL(t)), entropy purity EP(t), and negativity entanglement NE(t) are shown in (**a**) and in (**b**) with the same parameters as Figure 1a and Figure 1b, respectively, but for $\delta_{A}=\delta_{B}=2\lambda$.

Figure 2 displays the dynamics of two-charge qubits initially prepared in a maximally correlated state coupled to CEC and EC configurations in the absence of intrinsic decoherence. Here, we aim to analyze the entropic uncertainty relations UL(t), UR(t), entropy EP(t) and negativity NE(t). The detuning effects between the charge-qubits and coupled cavities are introduced, $\delta_{A}=\delta_{B}=2\lambda$. The appearance of the detuning effects is clear, and the revival character in the functions UL(t), UR(t), EP(t) and NE(t) is increased. This can be viewed as an improvement in the memory properties of coherent fields with detuning preventing permanent entanglement loss by avoiding maximal entropic uncertainty and entropy in the system, which is consistent with the results published in [46]. In addition, the revival rates are completely different for the resonant ($\delta_{i}=0$) and non-resonant case examined in Figure 1 and Figure 2 for the CEC configuration. For $\delta_{A}=\delta_{B}=0$, the preservation intervals of nonclassical correlations are extended but with fewer revivals; for $\delta_{A}=\delta_{B}=2\lambda$, the revivals increased, but the preservation intervals decrease in CEC configuration. In agreement, the EC configuration shows more revivals and less stable time in the current case as compared to the results in Figure 1. The revival rate can be traced back to the results obtained for a system of two atoms coupled with a single cavity field [53], and two qubits coupled with a mediated cavity field [54]. According to our results, the EC configuration performs better than the CEC configuration in terms of purity and nonclassical correlations because of the enhanced memory features of the coherent fields, when the non-zero detuning is considered. This contradicts the results illustrated in Figure 1, which shows that the CEC configuration performed better in the resonance. The dynamical behaviours of UL(t) and UR(t) differ from that observed in the previous CEC and EC configuration cases. The UL(t) function reaches a higher maximum level in CEC configuration when compared to the UR(t) function. However, there is no sign of periodic dynamics in the quantum memory-assisted entropic uncertainty relation as observed in [55]. On the contrary, the average maximum levels of the UR(t) function seem higher compared to the UL(t) in the EC configuration. Increasing the detuning can significantly improve the interaction between the charge-qubits and coupled fields, resulting in faster and greater information exchange and related characteristics.

**Figure 3:**
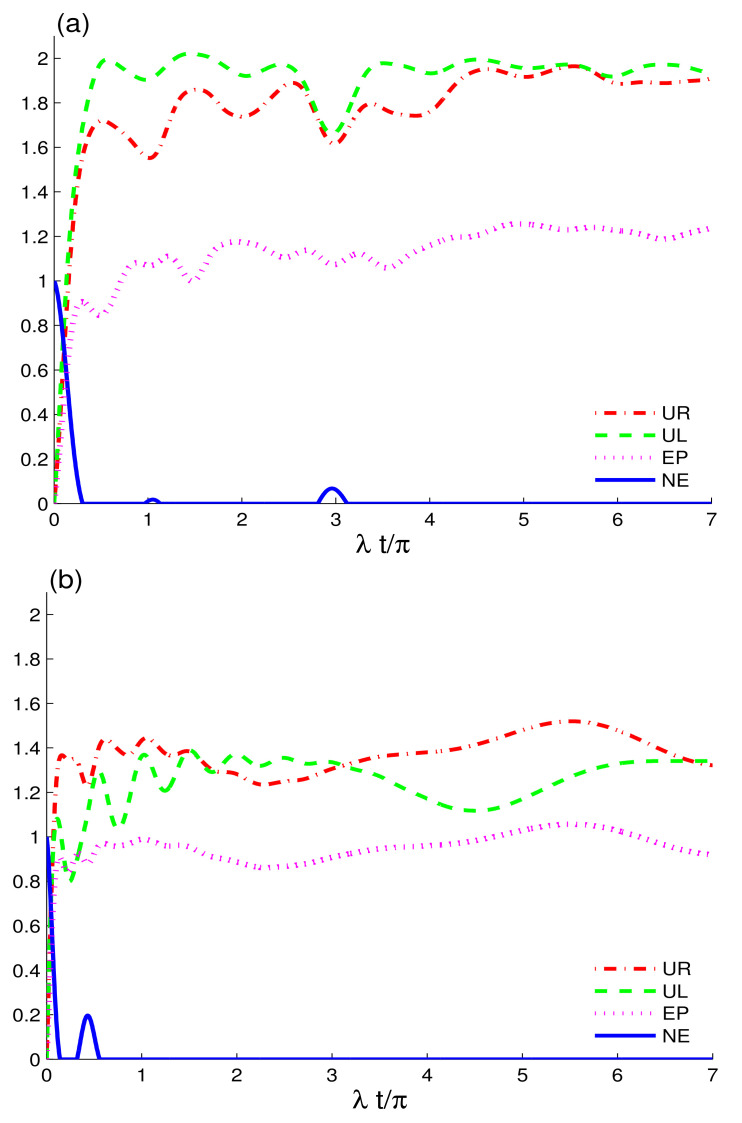
Dynamics of the quantum memory-assisted entropic uncertainty (UR(t) and UL(t)), entropy purity EP(t), and negativity entanglement NE(t) are shown in (**a**) and in (**b**) with the same parameters as Figure 1a and Figure 2a, respectively, for CEC configuration but in the presence of the intrinsic decoherence γ=0.06λ.

Figure 3 displays the dynamics of entropic uncertainty, related lower bound, entropy, and entanglement using UL(t), UR(t), EP(t) and NE(t) functions, when two non-interacting charge-qubits are exposed to CEC configuration. The effects of the intrinsic decoherence on the initial nonclassical correlations and quantum memory are also analyzed. The UL(t), UR(t), EP(t) and NE(t) functions behave differently because of the intrinsic decoherence; see Figure 1, Figure 2 and Figure 3. Nonclassical correlations and entropy, on the other hand, grow in opposing directions, demonstrating that, as the system’s entropy increases, the entanglement diminishes—as a result guaranteeing that entropic increases in a system result in mixedness and a decrease in field memory features and entanglement. In addition, the revivals in correlations between the two-charge-qubits are reduced. The intrinsic decoherence induces less interaction exchange between charge-qubits and the coupled CEC field, thus causing larger irreversible information decay. In Figure 3a, we set the detuning $\delta_{A}=\delta_{B}=0$ and in Figure 3b $\delta_{A}=\delta_{B}=2\lambda$. In both cases, the preservation and fluctuations in the state’s correlations are negligible; except in the latter situation, correlations only revive. The reduced revival feature in the current case can be traced back to the increased decoherence. We conclude that the presence of intrinsic decoherence in CEC fields plays a major role in causing correlations losses, which is in concordance with the results obtained for different quantum systems’ correlations under intrinsic decoherence [44]. Furthermore, due to the intrinsic decoherence, the detuning effects on the qubits diminish, resulting in no apparent revivals or extended correlations’ preservation. See Figure 1 for an example of the dynamical behaviour of the charge-quits with detuning versus that without detuning. Despite this, in the CEC configuration, detuning the charge-qubits can be used to reduce the loss of nonclassical correlations and purity of the system. We observe that, as entropy lowers, the mixedness in the system decreases, resulting in an increase in the purity of the state and related recovery of quantum correlations and memory properties in CEC fields. By comparing the final maximum values of the UL(t), UR(t), and EP(t) measures, this statement can be justified. The NE(t) measure has fewer revivals than the UL(t), UR(t), and EP(t). This demonstrates the dominance of the UL(t), UR(t), and EP(t) measures in encountering revivals when compared to NE(t). On the other hand, in the presence of detuning, the UR(t) reaches higher maximum levels than the UL(t), which contradicts the majority of the previous studies [55,56].

**Figure 4:**
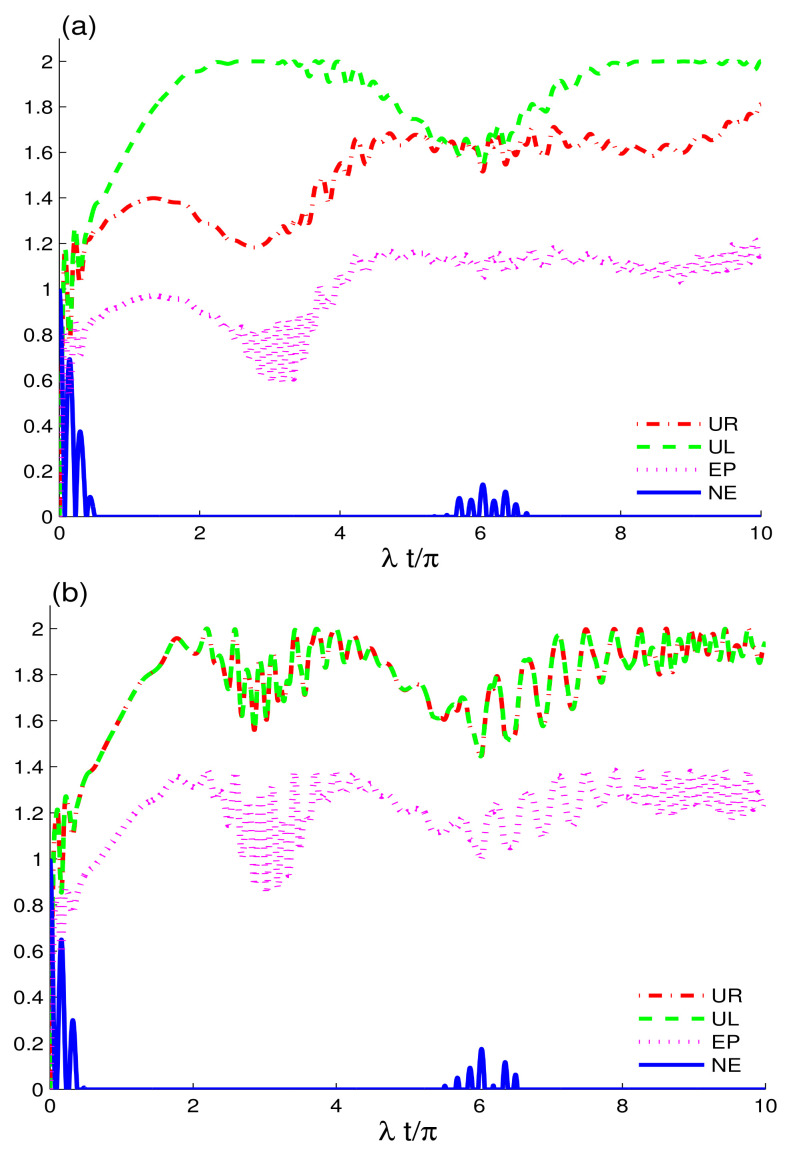
Dynamics of the quantum memory-assisted entropic uncertainty (UR(t) and UL(t)), entropy purity EP(t), and negativity entanglement NE(t) are shown for the initial maximally correlated state in the absence of the decoherence γ=0 and detunings $\delta_{i}=0$. When the cavities are initially prepared as CEC configuration in (**a**) and in EC in (**b**) for large coherent strengths $|\alpha_{A}{|}^{2}=8$ and $|\alpha_{B}{|}^{2}=10$.

Figure 4 displays the dynamics of quantum memory-assisted entropic uncertainty, entropy and entanglement using UL(t), UR(t), EP(t) and NE(t) quantifiers. In the absence of decoherence and detuning, we set $|\alpha_{A}{|}^{2}=8$ and $|\alpha_{B}{|}^{2}=10$ to focus on the effects of large coherent cavity field strengths. The enhanced coherence intensity strengths of the fields induce a qualitative shift in the dynamics of the UR(t), UL(t), EP(t), and NE(t). As demonstrated in Figure 1, when the coherent intensity is lowered to a minimum, the nonclassical correlations and purity remain better preserved in the state than when the coherent intensity is augmented. This is because, as the cavities become more coherent, leading the correlations to decay quickly. Thus, it is worth noting that the memory features of the coupled coherent fields are primarily concerned with the coherence strengths, and, as this strength grows, the memory characteristics of the fields become less robust, resulting in a more permanent decay of information in the two charge qubits. The CEC configuration remains more favourable for nonclassical correlations and coherence preservation during the early interaction time. The EC configuration exhibits slightly greater decay initially. The results for both CEC and EC fields at higher coherence strengths are opposite to those obtained for the identical configurations at low coherent strengths, as shown in Figure 1. The CEC configuration for high coherence strengths outperforms the EC configuration. As can be seen, the maximum levels of the UL(t), UR(t) and EP(t) functions in the EC field are larger, leading to greater entropic uncertainty, entanglement and purity loss. Furthermore, the entropic uncertainty functions revealed a higher increase in entropic uncertainty in two qubits driven by the Dzyaloshinskii–Moriya interaction described [57]. Entropic relations and entropy rise quickly for stronger coherent intensity fields, leading to entanglement decay between two-charge qubits occurring faster. When compared to the CEC configuration, the function UR(t) exhibits a dominant dynamical behaviour under the influence of the EC configuration, which is consistent with Figure 2.

**Figure 5:**
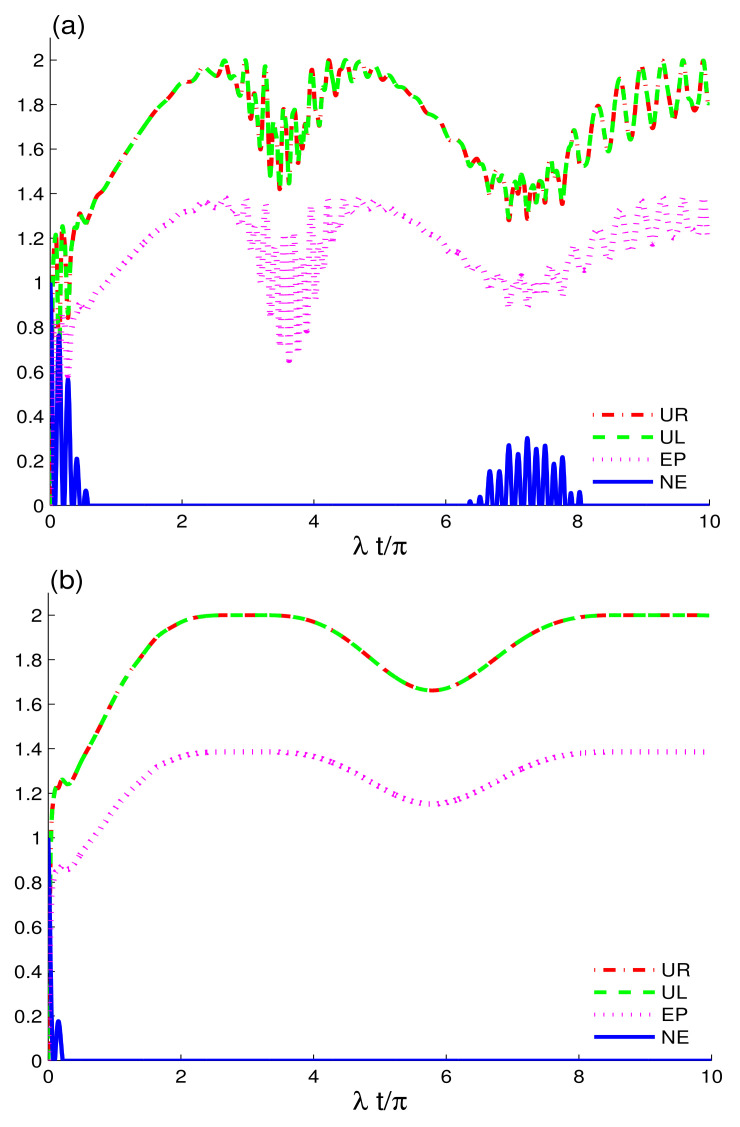
Dynamics of the quantum memory-assisted entropic uncertainty (UR(t) and UL(t)), entropy purity EP(t), and negativity entanglement NE(t) are shown as Figure 4b for EC configuration, but under the effects of the two-charge-qubit detunings $\delta_{A}=\delta_{B}=2\lambda$ in (**a**) and of the intrinsic decoherence γ=0.06λ in (**b**).

In Figure 5, we show the dynamics of entropic uncertainty relations, entropy purity, and negativity entanglement versus time for two-charge-qubits coupled to two independent cavities prepared in EC configuration. In this case, we consider two schemes: in Figure 5a, the two-qubit detuning is set to $\delta_{A}=\delta_{B}=3\lambda$, and, in Figure 5b, the intrinsic decoherence is set as: γ=0.06λ. In comparison to the case with detuning, the intrinsic decoherence effects cause the entropy functions to gradually increase, resulting in faster degradation of entanglement, purity, and memory properties of the coherent fields. When decoherence arises, the revival character of the two-qubit correlations appears to be totally repressed, and this agrees with the results obtained for different configurations under intrinsic decoherence explored in [44]. The entanglement exhibits numerous rebirths in the period 7.5≤λt/π≤9.5, avoiding total correlations losses in the off-resonant case. In agreement, the entropic relations and entropy display stronger revivals than those reported in the presence of the intrinsic decoherence, meaning that the memory properties of the coherent fields are very fragile to the related intrinsic decoherence effects. Thus, in an EC configuration, detuning the charge-qubits can improve nonclassical correlations and purity preservation and avoid a total loss. Besides the improvement in the memory properties of the fields, detuning promotes information exchange between the system and fields in some quantum systems [58,59], where interaction between the system and fields is essential.

From the above results, we deduce that the entropic relation functions UL(t) and UR(t) are sensitive to smaller changes in CEC and EC fields. Both detect smaller changes in the entropic uncertainty in a two-qubit system coupled to CEC and EC configurations, such as revivals or related decline and rise. The EP(t) measure shows robust revivals of entropy at relevant intervals and aligns well with the entropic uncertainty functions. The entropic uncertainty relations and entropy maximums coincide. In contrast, the negativity entanglement measure NE(t) has fewer revivals than UL(t), UR(t) and EP(t). As a result, it failed to show the fields’ true revival character. Furthermore, the relative memory properties of the fields rely not only on their parameters but also on the type of fields involved. We show that the memory characteristics of the CEC and EC fields do not preserve nonlocal correlations in the same way. In accordance with Ref. [46], we came to the conclusion that the memory properties of the coherent coupled fields are substantially reliant upon the associated Markovian proprieties. As shown in Figure 1, Figure 2, Figure 3, Figure 4 and Figure 5, we find that, when the Markovian character occurs, quantum correlations are preserved, and hence memory properties are preserved. As a result, we establish that the Markovian nature of current fields, associated quantum correlations preservation, and entropic changes with memory features are all intrinsically connected.

## 5. Conclusions

Two charge qubits inside two cavities have been explored in a system that is initially in a non-symmetric Bell state. Each qubit independently interacts with its cavity. Quantifying the amount of quantum memory-assisted entropic uncertainty, entropy, and negativity as well as the related revivals has been the main focus of this paper. The coherent fields are considered in two separate schemes: coherent-even coherent and even coherent state configurations. In the coherent-even coherent case, the first cavity is initially in a coherent state while the second cavity is prepared in an even coherent state. In the second case, both cavities are prepared in even coherent states. We show that the coherent configurations efficiently preserve nonclassical correlations for defined interaction times. On the other hand, the cavity features are critical for the preservation period as well as the dynamics of nonclassical correlation and coherence. Even coherent cavity fields perform better in terms of preserving nonclassical correlations and memory properties when the system is in resonance. Mixed coherent and even coherent state fields, on the other hand, are suitable for nonclassical correlations and purity preservation when detuning is increased. Furthermore, when the amplitude of intrinsic decoherence grows, nonclassical correlations decay faster, limiting the exchange of information between the state and the coupled-cavity fields. In the absence of intrinsic decoherence, the cavity field, on the other hand, can be useful to keep quantum phenomena in the states for longer periods. Detuning has a major influence on the charge qubit-field correlations’ preservation and dynamics. The interaction and interchange of information between the system and fields intensify when the detuning of the qubits rises. The revival of the nonlinear correlations reduces as the fields’ coherence strength decreases. When intrinsic decoherence is neglected and field detuning and coherence strength are set higher, the coherent-even coherent and even coherent configurations could be used to design longer nonlocal correlations and coherence in charge qubits. We have also observed that the entropic uncertainty is more vulnerable to external field effects as compared to the entropic uncertainty bound and entropy. Unlike entropic uncertainty, related lower bounds, and entropy, the negativity exhibits the least detection of entanglement losses and gains in two-charge qubits. 

## Data Availability

Not applicable.
